# Beyond Junk-Variable Tandem Repeats as Facilitators of Rapid Evolution of Regulatory and Coding Sequences

**DOI:** 10.3390/genes3030461

**Published:** 2012-07-26

**Authors:** Rita Gemayel, Janice Cho, Steven Boeynaems, Kevin J. Verstrepen

**Affiliations:** 1 Laboratory for Systems Biology, VIB, Gaston Geenslaan 1, B-3001 Heverlee, Belgium; rita.gemayel@biw.vib-kuleuven.be (R.G.); jacho@students.pitzer.edu (J.C.); steven.boeynaems@student.kuleuven.be (S.B.); 2 Laboratory for Genetics and Genomics, Centre of Microbial and Plant Genetics (CMPG), KULeuven, Gaston Geenslaan 1, B-3001 Heverlee, Belgium

**Keywords:** microsatellites, repeats, evolvability, phenotype, polyglutamine

## Abstract

Copy Number Variations (CNVs) and Single Nucleotide Polymorphisms (SNPs) have been the major focus of most large-scale comparative genomics studies to date. Here, we discuss a third, largely ignored, type of genetic variation, namely changes in tandem repeat number. Historically, tandem repeats have been designated as non functional “junk” DNA, mostly as a result of their highly unstable nature. With the exception of tandem repeats involved in human neurodegenerative diseases, repeat variation was often believed to be neutral with no phenotypic consequences. Recent studies, however, have shown that as many as 10% to 20% of coding and regulatory sequences in eukaryotes contain an unstable repeat tract. Contrary to initial suggestions, tandem repeat variation can have useful phenotypic consequences. Examples include rapid variation in microbial cell surface, tuning of internal molecular clocks in flies and the dynamic morphological plasticity in mammals. As such, tandem repeats can be useful functional elements that facilitate evolvability and rapid adaptation.

## 1. Introduction

### 1.1. Tandem Repeats—Definitions and Characteristics

The scientific community has previously dismissed repetitive regions as nonfunctional, “junk” DNA. However, recent evidence with the assistance of whole genome sequencing illuminates the significant role repeats might play in genomes. In the 1960s, scientists identified these repetitive elements as the explanation for the negative correlation between an organism’s phenotypic complexity and its genome size [1]. For instance, repeats constitute almost 46% of the entire human genome and prokaryotic genomes contain roughly 10% repetitive regions, a significant amount considering their small sizes [2]. 

**Figure 1:**
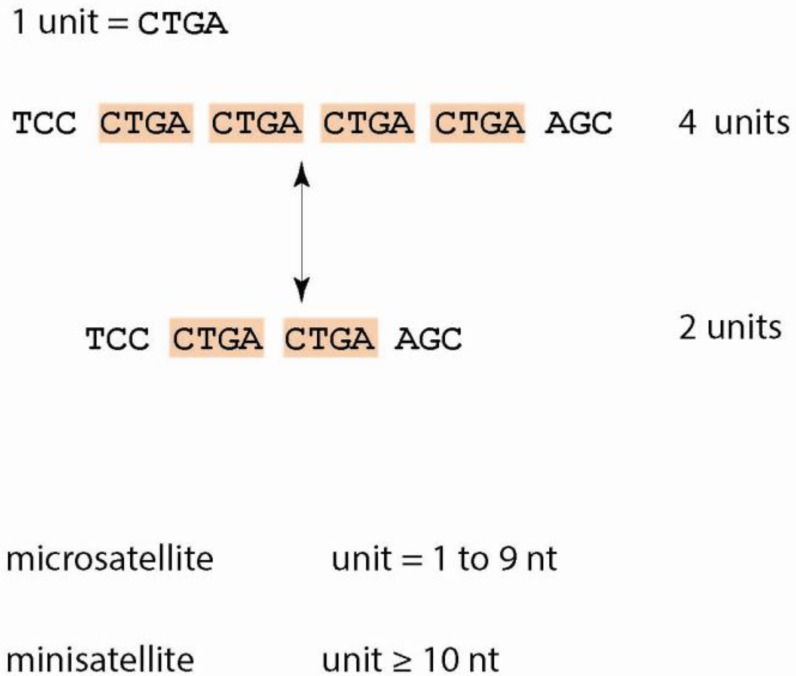
Main definitions and characteristics of tandem repeats (TRs). TRs are unstable due to frequent changes in the number of repeat units. TRs with short unit length are also called microsatellites and those with long units are called minisatellites.

Two categories of repetitive regions exist—interspersed repeats and tandem repeats (TRs). Interspersed repeats, the more predominant type of repeat, are remnants of transposons dispersed throughout the genome. Such elements are responsible for the diverse array of genome sizes amongst various species [3]. On the other hand, TRs are repetitive DNA, which exist directly adjacent, or in tandem, to one another (Figure 1). TRs are often referred to as satellite DNA because they were first identified as sequences constituting the second or “satellite” band after subjection through density-gradient centrifugation [4].

On the basis of unit length (unit = repeated sequence of DNA—see Figure 1), TRs are further divided into two subcategories—microsatellites and minisatellites. Microsatellites, or simple sequence repeats (SSRs), are short TRs with unit length between one to ten nucleotides. Minisatellites are TRs with unit length larger than ten nucleotides. For the purposes of this review, we limit our discussion to tandem repeats.

### 1.2. Instability of Repeats

Tandem repeats are evolutionarily pertinent due to their instability; they mutate at rates between 10^−3^ and 10^−6^ per cellular generation (*i.e.*, 1 to 10 orders of magnitude greater than point mutations) [5]. Repeat polymorphisms usually occur from the addition or deletion of repeat units, rather than nucleotide substitutions. For instance, in a CTGA tract, most mutations occur by the addition or deletion of an entire CTGA unit as opposed to rare cases in which only a part of the repeat unit is altered (e.g., deletion of two nucleotides GA) (Figure 1).

Two major models have been proposed to explain TR expansions and contraction: strand-slippage replication and recombination. Strand-slippage replication (slipped-strand mispairing or DNA slippage) is a DNA replication error by which mispairing occurs between the template and nascent strands. As such, the template strand can loop out, causing contraction; the nascent strand can also loop out, leading to repeat expansion. Recombination events, such as unequal crossing over and gene conversion may additionally lead to contractions and expansions of TR sequences [5,6].

Various studies suggest alternative explanations for mutation events. Some studies implicate that strand-slippage replication dominates micro- and minisatellite instability [7]; others indicate that strand-slippage replication generally associates with microsatellite instability whereas recombination effects dominate minisatellite instability [8]. However, the precise mechanism by which repeat mutations occur remains unclear.

### 1.3. Factors Influencing Repeat Instability

The frequency of repeat mutation depends on repeat tract: the longer and purer the repeat, the higher the mutation frequency whereas the shorter and less pure the repeat, the lower the mutation frequency. Even a few impurities drastically reduce mutability of repeat regions [9], indicating that impure repeats demonstrate far greater stability than pure repeats. One study showcases that even a single variant repeat in an otherwise pure poly GT tract increases its stability fivefold [10]. According to Legendre *et al.* 2007, the number of repeat units is the most important determinant of TR stability, above purity and repeat unit length. Base composition also affects repeat stability: polyC and polyG tracts are more unstable than polyA and polyT tracts [11].

Cellular processes may additionally influence TR mutation rates. It has been shown that increased transcription rates can enhance repeat instability; in yeast, transcription of a poly GT tract destabilizes the repeat region four-to-ninefold with higher levels of transcription causing an additional destabilizing effect of two- to three-fold [12].

External factors may also significantly influence repeat stability. Classic examples include CT repeat contraction upon fungal infection [13] and repeats in bacteria that undergo frequent adaptive, environmentally stimulated mutations [14]. Likewise, inhibition of Hsp90 (a protein generally involved in responding to severe environmental changes) increases CAG repeat contraction by tenfold, while not affecting the rate of point mutations, illustrating how environmental stresses may trigger TR mutations [15].

### 1.4. In Search of Repeats

Whole genome sequencing has enlightened researchers on the prevalence of repetitive regions in the genome. A comprehensive survey of the various software packages and algorithms for TR detection has been previously published [16]. Certain obstacles remain in our quest to understand more about TRs. One significant issue arises due to the tedious nature of TR identification. Despite the availability of numerous algorithms for identifying TRs (*i.e.*, Tandem Repeat Finder, Sputnik, TROLL) which define certain requirements for TRs based on the number of repeated units, purity of repeats, as well as length of repeat region, no definite guideline exists and algorithms vary from one to another. Therefore, it proves to be quite difficult in assigning a proper cutoff score and an overestimation of TR regions may ensue without the enforcement of stringent guidelines for TR identification.

Certain measures may be taken to prevent such scenarios (e.g., the shuffle method). Multiple, randomly shuffled sequences of similar size and composition as the sequence of interest are selected and subjected to a TR-finding program. These sequences are searched for TRs using various cutoff scores. The average number of TRs identified in these scrambled sequences then serves as a value of background noise produced by chance occurrence of TRs (for more details, see [17]. A sequence mining tool such as SERV identifies TRs in various DNA sequences and predicts a “variability score” of each repeat based on its purity, unit size, and number of repeat units [9]. These procedures allow for proper identification of significant, variable TRs rather than insignificant “background noise”.

### 1.5. Location of Repeats in Genomes: Coding and Regulatory Regions

Though repeats are overabundant in gene deserts, whole genome sequencing capabilities provide concrete evidence for the prevalence of repeats in coding regions as well. This is exemplified by the human genome; 17% of genes contain repeats in open reading frames and such values are comparable in other species as well [17,18] Microsatellites (<10 nt) are specifically enriched in regulatory genes that encode for transcription factors, DNA-RNA binding proteins, and chromatin modifiers [19]; minisatellites (>10) are prevalent in genes encoding extracellular or cell-surface genes [5]. 

Unlike repeats in noncoding regions, the high frequency of tri- and hexanucleotide repeats (units of 3 or 6 nucleotides) found in coding regions suggests a selective pressure against frameshift mutations [20]. For instance, of the 1 in 20 human genes that were found to contain TR polymorphisms, only approximately 1% of those genes exhibited frameshift mutations [21]. Exceptions apply to prokaryotes in which frameshift mutations act as a beneficial mechanism (to be further discussed later). 

In coding regions, repeats exhibit biases for specific amino acids. Glutamine, arginine, glutamate, alanine, and serine residues largely dominate eukaryotic genomes [22] and [23] whereas serine, glycine, alanine, and proline residues dominate prokaryotic genomes [24]. Interestingly enough, hydrophobic residues such as isoleucine, methionine, and tryptophan residues are generally absent, indicating that preferences for certain residues are not random but stem from the functional role of repeats.

In many instances, repeat mutations, especially in gene deserts, have neutral effects and remain undetected. Cases like these may have contributed to the previous notion that repeats were nonfunctional junk or selfish DNA [25,26,27]. However, repeat instability in coding regions, promoters, and other regulatory regions may eventually proceed to alter gene function. In this review, we highlight the studies that implicate variable TRs with changes in gene function, starting by TRs located within regulatory regions (promoters and other regulatory sequences) and ending with studies on intragenic (*i.e.*, coding) repeats.

## 2. Repeats in Non-Coding Regions

Phenotypic evolution has been mostly attributed to changes in gene expression and regulation due to variation in (non-coding) *cis*-regulatory elements [28,29]. Upon analysis of the human genome, Rockman and Wray, found a higher heterozygosity in *cis*-regulatory sites than in coding sequences in part, due to the high prevalence of TRs in regulatory regions, particularly the AC dinucleotide repeat [30]. The authors suggest that these repeats contribute significantly to variable gene expression in humans and are thus sources of rapidly evolving, heritable phenotypic variation. A similar analysis in the yeast *Saccharomyces cerevisiae* indicates that about 25% of all promoters contain TRs. Variation in these repeats is associated with variable gene expression within and between yeast species [31] .

Additional studies uncover a link between variable TRs in promoters and changes in gene expression and/or function. This suggests that instead of being neutral sequences without a functional value, TRs may actually be significant contributors to functional variation in gene expression. In the following section, we report the studies that reveal a link between variable TRs and changes in gene expression. We focus particularly on studies which reveal the mechanism through which repeats in non-coding regions affect downstream gene expression. A summary of numerous correlation studies, in which gene expression variation has only been associated with variable promoter TRs, can be found in the following reference [17].

## 3. Promoter Evolution Through Tandem Repeat Variation

The high prevalence of TRs in regulatory regions, in many organisms, suggests that repeat variation might be a common, evolutionarily conserved, mechanism regulating gene expression. A large number of studies report correlations between TRs and changes in gene expression [17]. Other studies, in addition, provide a solid mechanistic explanation as to how repeat variation leads to changes in downstream gene expression. These studies will be discussed in greater detail in this section. 

### 3.1. Tandemly Repeated Transcription Factor Binding Sites

Variable TRs in promoters can affect gene expression by altering the number of transcription factor binding sites. Frequent TR variability of repeat-containing transcription factor binding sites results in changes in the number of binding sites. An example of such a mechanism was first uncovered in pathogenic bacterial species. In *Neisseria meningitidis*, changes in the number of TAAA repeats located in the *nadA* promoter induces variable expression of the NadA adhesin protein. This highly frequent switching of phenotypic outcome, also referred to as phase variation, is mediated by the loss or gain of binding sites for the regulatory protein IHF [32]. A second, repeat-dependent, level of control on the same *nadA* promoter is discussed in the following paragraph. Rapid phenotypic switching mediated by intragenic (*i.e.*, coding) repeats will be discussed in a later section of this review. 

Changes in transcription factor binding sites as a result of unstable TRs have also been described in eukaryotes. A variable TCC repeat in the promoter region of the human epidermal growth factor receptor (a proto-oncogene) induces changes in gene expression by altering the number of binding sites for the transcriptional regulator Sp1 [33]. A change of one unit in a 61-bp repeat of the human reduced folate carrier gene promoter induces a significant change in gene expression. This repeat is polymorphic in the human population and binds the transcription factors Sp1 and AP2 *in vitro* [34]. Though these display a few examples of the role of TRs in transcription factor binding sites, a screen of polymorphic TRs located in a 10-kb region upstream of the transcription start sites in the human genome reveals that there are many more TRs that bind regulatory proteins and thus act as *cis*-regulatory elements [35].

### 3.2. Variable Repeats Induce Variable Spacing Between Functional Promoter Elements

**Figure 2:**
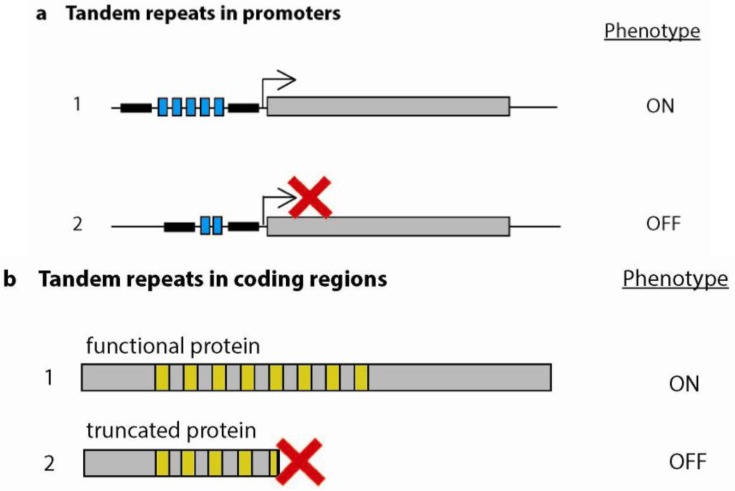
Phase variation mediated by variable TRs. Variable TRs control phase variation to allow pathogenic bacteria to rapidly interchange between phenotypes. (**a**) Variable tandem repeats in promoter regions control gene expression. A certain repeat number allows for RNA polymerase binding in promoter regions enabling proper gene expression (confers the ON phenotype) whereas TR truncation disables RNA polymerase binding leading to blocked expression (confers the OFF phenotype); (**b**) Variable TRs located in coding regions produce either a fully-functioning protein (confers the ON phenotype) or truncated, protein (confers the OFF phenotype).

Variable TRs in promoters not only affect the number of transcription factor binding sites, but can also induce changes in spacing between critical promoter elements (Figure 2). In the *nadA* promoter of *N. meningitidis* (see above), changes in the number of TAAA repeats also induce changes in spacing between distal and proximal binding sites (flanking the TAAA repeat) for the NadR repressor protein [36]. Additionally, the transcript level of the *N. meningitidis* membrane protein PorA correlates with the number of guanidine residues within the -10 and -35 spacer region in the upstream promoter. A mere loss or gain of one nucleotide in this polyGuanidine TR leads to significant changes in PorA transcription [37]. Similarly, in *Haemophilus influenzae*, promoter TRs modulate the expression of genes involved in the formation of adhesive appendages or fimbriae. Variation in a TA dinucleotide repeat in the bidirectional promoter of two genes *hifA* and *hifB* alters the critical spacing between the -10 and -35 consensus sequence, thus affecting the bidirectional transcription initiation of both genes [38]. Several phase variable outer membrane proteins, whose expression is attributed to similar promoter TRs, have also been described in *Bordetella pertussis* [39], and *Mycoplasma hyorhinis* [40].

Genome-wide sequencing suggests there are many more pathogenic bacteria, than the ones mentioned above, in which tandem repeat-mediated phase variation is operable. In fact, more than 30 *N. meningitidis* genes are expected to be phase variable as a result of unstable TRs [41]. 

It seems that this form of gene expression regulation (changes in spacing between functional promoter elements) is common among several pathogenic bacterial species. This rapid phenotypic switching, results in a heterogeneous bacterial population in which some individuals are better suited to environmental changes (e.g., attack by the immune system) and consequently have better chances of survival.

### 3.3. Dynamic Chromatin Structure and Nucleosome Positioning

DNA is intricately organized in the nucleus and packaged into structures called nucleosomes, in which DNA is wrapped around an octamer of four core histones, to form chromatin. Chromatin structure is highly dynamic and has large implications in the regulation of gene expression, especially since histone complexes affect accessibility of transcription factors to DNA. 

**Figure 3:**
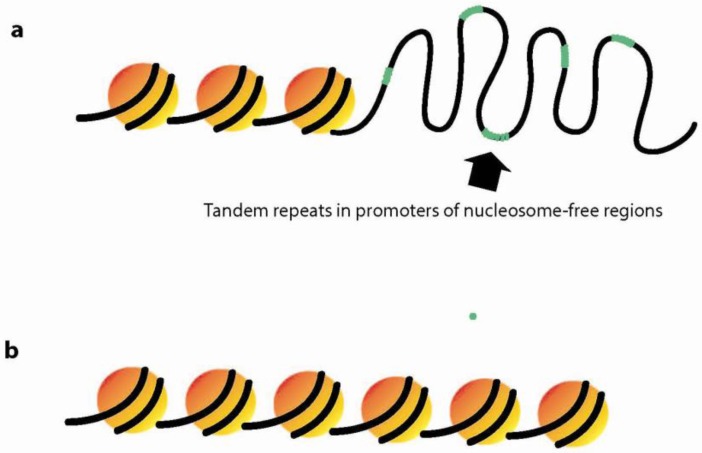
TRs mediate dynamic chromatin structure. (**a**) TR enrichment in promoter regions is nucleosome-inhibiting and allows for an open chromatin structure; (**b**) Nucleosome-dense regions lack TRs in promoter regions of the genome.

Several studies indicate a possible role for repetitive amino acid tracts in mediating effects on chromatin structure. A number of authors demonstrate the importance of polyA and polyT tracts as constituents of promoter regions that inhibit nucleosome formation [42,43,44]. Additionally, Vinces *et al*. illustrates TR enrichment in nucleosome-free regions of yeast promoter regions and supports this finding with preliminary observations in human promoters (Figure 3). Mutants were made in repeat-containing promoters of several yeast genes and found to affect gene expression and nucleosome positioning. This study unveils that TRs behave as nucleosome-inhibiting sequences that allow for an open chromatin structure [31]. The authors also find that approximately 25% of yeast genes contain TRs, which has strong implications for the importance of TRs as crucial components in genomes of higher-order eukaryotes that regulate DNA structure and facilitate DNA melting. The following section further explores the role of TRs in the regulation of DNA structure.

### 3.4. Intrinsic Properties of Tandem Repeats Affect DNA Structure

Though DNA is normally found as B-DNA (right-handed helical conformation), in rare cases, it may exist as Z-DNA (left-handed helical conformation). This conformation is drastically different from the normal B-DNA, that it might block access of transcription factors to DNA. Several studies suggest a role for TRs as elements that assist in Z-DNA formation. Alternating purines and pyrimidines, which favor the Z conformation, are found as TR sequences in the genome. Authors Naylor and Clark find evidence for a CA repeat lying upstream of the rat prolactin gene that enables Z-DNA formation. Additionally, this repeat inhibits gene transcription by potentially deterring transcriptional efficacy of RNA polymerase II [45]. Specific Z-DNA-binding proteins also influence Z-DNA gene expression as exemplified by the effect of double stranded RNA adenosine deaminase (ADAR1) proteins that bind promoter Z-DNA to activate gene expression [46].The structure of DNA greatly influences gene expression and these studies allude to the possible importance of TRs for DNA structure.

## 4. Repeats in Introns and Untranslated Regions

Functional roles for TRs present in introns and untranslated regions have been mostly uncovered by studies investigating the pathogenic mechanisms involved in neurodegenerative diseases. These studies provide insight into how variable intergenic TRs can act as modulators of gene expression. In this section, we focus on such studies and highlight the mechanism through which variable TRs in introns and untranslated regions can lead to changes in gene expression. 

### 4.1. Recruitment of RNA Binding Proteins

Tandem repeats in introns and untranslated regions can influence gene expression by modulating the activity of RNA-binding proteins. The mechanistic role of TRs in recruiting RNA-binding proteins has been proposed to explain the overlapping clinical features of two genetically unrelated neurological diseases. Expansion of a CTG repeat in the 3' UTR of the *DMPK* gene causes myotonic dystrophy type 1 (DM1) and the expansion of a CCTG repeat in intron 1 of the *ZNF9* gene causes myotonic dystrophy type 2 (DM2). The expanded RNAs adopting a stable hairpin structure, bind to and alter the activity of RNA processing proteins CUG-BP1 and MNBL [47]. This leads to aberrant splicing of numerous transcripts (*i.e.*, downstream targets) involved in different cellular processes. 

### 4.2. Variable mRNA Splicing Patterns

**Figure 4:**
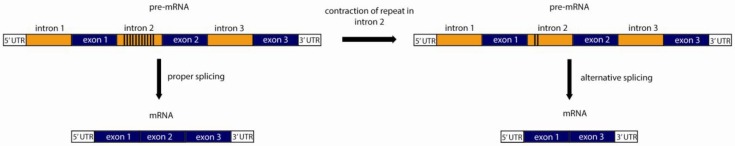
Variable intronic TRs regulate mRNA splicing. (**a**) A variable TR in intron 2 in the pre-mRNA transcript enables proper splicing to form a fully-functioning, normal mRNA transcript; (**b**) Shortening of intron 2 repeat results in an alternatively spliced (non-productive) transcript missing exon 2, which does not produce any functional protein (from reference [48]).

A study investigating the link between expression of the Bromodomain-Containing Gene (*BRD2*) and epilepsy in humans uncovered the role of variable intronic repeats in regulating gene expression through alternative mRNA splicing pattern. In intron 2 of the *BRD2* gene, a polymorphic GT repeat is strongly associated with epilepsy. Gradual shortening from 13 GT repeats to 1 GT repeat results in a higher proportion of (non-productive) alternatively spliced *BRD2* transcripts over normal transcripts. These alternative mRNAs produce non-functional BRD2 proteins [48]. Similarly, a change from 11 CA repeats to 13 repeats in an intron of the cystic fibrosis transmembrane regulator results in higher levels of a non-functional transcript (missing exon 9) [49]. These examples illustrate how intronic dinucleotide repeats alter splicing patterns, ultimately affecting translation and protein synthesis (Figure 4). 

### 4.3. How Can Variable Tandem Repeats in Regulatory Regions Promote Evolvability?

The studies described in this section indicate that variable TRs in regulatory regions can strongly impact gene expression and/or function through various mechanisms. The high prevalence of these repeats in diverse genomes suggest that this type of gene expression regulation (*i.e.*, through variation in TR number) might be a common and conserved regulatory mechanism. The central question that remains is how can TR variability promote evolvability? And what are the effects on an organismal level? 

The studies on bacterial phase variation clearly demonstrate how repeat variation (in promoters and coding regions—see further) enable phenotypic evolution, ultimately impacting the population; rapid changes in TRs create phenotypic variants within a population, leading to fast adaptation (e.g., to the immune system) and increased survival. 

In eukaryotes, a recent study provides experimental evidence on the role of TRs as drivers of rapid evolution. Vinces *et al.* reports that yeast promoters containing TRs have evolved faster since their expression patterns are more divergent between and within yeast strains compared to promoters without repeats [31]. Promoter-containing repeats fused to a fluorescent marker to facilitate selection for higher gene expression provide experimental evidence for repeat-driven rapid evolution after a few rounds of selection: repeat-containing promoters yield variants with higher expression that are specifically linked to changes in promoter repeat number; repeat-less promoters do not show any changes in gene expression during the selection process [31]. This study firmly establishes that several promoter repeats generate expression variability at high rates, allowing for rapid evolution of gene expression.

In complex organisms, such studies are difficult to undertake, though some reports indicate that this might be the case for particular genes. The promoter of the *MMP3* gene contains a variable polyT tract that is rapidly evolving in the primate lineage. In humans, variation in this repeat results in variable expression of the *MMP3* gene, associated with risk of heart disease: the 5T allele results in higher *MMP3* expression and is associated with myocardial infarction and aneurysm, whereas the 6T allele drives a lower *MMP3* expression and is associated with coronary artery disease [50]. In European populations, the 5T allele seems to be under positive selection in comparison to other parts of the world. This highly variable repeat seems to drive the rapid evolution of the *MMP3* gene expression and based on its associated phenotype in humans, it clearly exhibits a functional value.

## 5. Repeats in Coding Regions

Tandem repeats in coding regions are mostly known for their association with a number of human neurodegenerative diseases. Yet their abundance in functional regions of the genome and their enrichment in certain gene categories, suggest that repeats in coding sequences might play a beneficial role. In this section, we review studies revealing functionally beneficial roles of long (minisatellite) and short (microsatellite) coding repeats.

### 5.1. Minisatellites—Functional Variability in Cell Surface Proteins

Minisatellites are enriched in cell surface genes and conserved through evolution from yeast to humans [9]. Various studies indicate that polymorphisms in these minisatellites in fact confer functional variability. 

**Figure 5:**
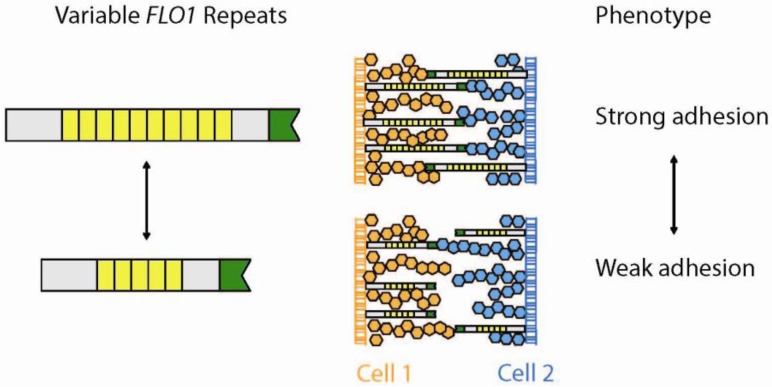
Variable coding TRs modulate cell to cell adhesion. The Flo1 protein binds cell wall polysaccharides (orange and blue hexagons) through its *N*-terminal domain (green) thus linking two yeast cells. Long *FLO1* repeats result in a longer Flo1 protein capable of stronger cell wall binding.

The *S. cerevisiae FLO1* gene encodes a cell-surface adhesin that mediates cell-cell and cell-surface adhesion. The gene contains a repeat tract consisting of tandemly repeated units of approximately 100 nt, which are variable across different yeast strains [5]. Phenotypic tests with isogenic strains differing only in their *FLO1* repeat length illustrate that the intensity of the adhesion directly correlates with the length of the repeat (*i.e.*, cells with longer *FLO1* repeats adhere better to each other) [5] (Figure 5).

*Flor* yeast strains form buoyant biofilms on the air/liquid interface, allowing them to evade oxygen-deprived environments. Fidalgo *et al*. report that the extension of a minisatellite in *FLO11*, another adhesin-coding gene, contributes to biofilm formation. The repeat tract consists of serine/threonine-rich units (~100 nt), prone to *O*-glycosylation. The addition of covalently linked sugar groups renders the adhesins, and subsequently the cell wall, hydrophobic. Thus, an increased number of repeats enhances buoyancy of yeast strains. Additionally, transformation of *S. cerevisiae* strains, that generally sediment, with *flor FLO11* repeat tract and promoter enable yeast cells to form buoyant biofilms [51].

Similar observations have also been made in the human pathogen *Candida albicans*. The *ALS* gene family encodes adhesins similar in structure to the baker’s yeast flocculins that mediate biofilm formation. The length of the central repeat domain correlates with adhesion propensity (*i.e.*, longer repeats generate more adhesive cells) [52]. Recombination events between the different *ALS* genes can generate cell-surface variability required to evade the host’s immune response [53]. 

### 5.2. Microsatellites—Beyond Diseases: A Greater Purpose for Coding Repeats

Trinucleotide repeats, in particular polyglutamine (polyQ) repeats, are involved in a number of human neurodegenerative diseases (e.g., Huntington’s disease, fragile X syndrome, spinocerebellar ataxia), with numerous studies illustrating the conferral of such diseases upon repeat expansions exceeding a particular threshold. Though TRs involved in human diseases will not be discussed extensively here, it is important to note that severity and time of onset of these neurodegenerative diseases depend on the repeat expansion: longer repeats correlate with earlier disease onset and more severe symptoms (for a review on the role of repeats in human diseases, refer to [47]).

Considering the little we know, such devastating effects of TR mutations have often overshadowed our knowledge of TRs. However, the predominance of repetitive DNA sequences in functional parts of the genomes, despite such a high propensity for mutations and apparent low information content, hint at a greater role for TRs. In fact, though the genetic code is degenerate, amino acid repeats tend to be coded by pure DNA repeats, illustrating the conservation of repetitive regions on the DNA level as well as the protein level. These unstable repeats, might be tolerated in some coding sequences because they hold the potential for the acquisition of a beneficial novel/improved function (e.g., novel protein-protein interactions,). TRs may not merely be “junk DNA” as was previously assumed. We will highlight key studies that provide experimental evidence for this hypothesis in the following section.

### 5.3. Repeats as Molecular Switches in Bacteria

Intragenic TRs can also mediate phase variation (see also section on repeats in non-coding regions). In the bacterium, *Neisseria gonorrhoeae*, repeat variations in the P. II family of cell surface genes allow for evasion of the host immune system [54]. Changes in the CTCTT pentameric microsatellite repeat in the coding region of the membrane signal peptide of cell surface genes, can either lead to correct or incorrect translation of proteins [55]. This switching between an ON and OFF state of the gene generates phenotypic variability in the population, providing individuals able to evade the host’s immune response (Figure 2).

Similar occurrences are seen in the *lic1* gene of *Haemophilus influenzae* (enables the addition of phosphorylcholine moieties to cell membrane lipopolysaccharides (LPS)), which contains a variable intragenic CAAT repeat. Formation of a complete, functioning Lic1 protein or a truncated mutant depends on variation of an intragenic CAAT repeat. Differences in LPA structures promote different bacterial variants enabling survival of the bacterium in the host [56]. 

Phase variable genes are not merely limited to cell-surface genes; recent evidence suggests that regulatory genes, such as those encoding DNA-methyltransferases (*mod* genes), are also phase variable. The m*odA* gene, in *Neisseria* and *Haemophilus* species, contains either an AGTC or AGCC repeat whose mutations directly alter the rate of phase variation [57]. The *modA* gene contains two potential start codons depending on repeat number but only the one that yields the longest transcript gives rise to a functional protein. Variation in *ModA* repeat number thus has consequences on the expression of its downstream targets that include outer membrane proteins, iron transporters and heat-shock proteins [57]. For instance, the OFF state of the *modA* gene leads to biofilm thickness and better survival rates of *N. gonorrhoeae* cells in human cervical epithelial cells [58]. The ability to engage in phase variation may provide a means to increase population fitness and facilitate rapid adaptation to a hostile environment.

Sequencing reveals the substantial presence of TRs in prokaryotic genomes, which suggests that rather than such an incidence being happenstance, some TRs are positively selected for rather than against [59]. Particularly in pathogenic bacteria, repeats in coding sequences are more favored at the *N*-terminus and depleted in the middle of the coding sequence [60]. This might increase the probability of frameshift mutations that facilitate phase variation. Thus, TRs may be useful elements that mediate phenotypic change enabling organisms to rapidly adapt to a changing environment. Ultimately, understanding phase variations in these prominent human pathogens may unveil new insights in combating significant health problems.

### 5.4. Significance of Polyglutamine Repeats

In humans, polyglutamine (polyQ) repeats have strong implications for neurodegenerative diseases [61]. In *Drosophila melanogaster*, polyQ repeats constitute one of the three most frequently found amino acid runs; the same can be said for the yeast genome [62]. Overall, polyQ tracts are abundant in the eukaryotic genome [24,62] and repeats encoding these residues are largely variable. 

Here we showcase several studies regarding the possible functional role of polyQ repeats, ranging from its involvement in morphological variation to protein regulation. 

#### 5.4.1. Polyglutamine Repeats Regulate Circadian Clocks

Organisms possess circadian clocks, or innate biological timing mechanisms that synchronize various cellular processes with the external environment. The typical circadian cycle, also referred to as a period, occurs over a span of 24 h. The circadian rhythm, cued by environmental factors (e.g., light intensity and temperature), is crucial to an organism’s survival and well-being; maximizing ideal circadian patterns may confer a fitness advantage.

In the species *Neurospora crassa*, intragenic CAG repeats control expression of the transcription factor white collar-1 (WC-1), responsible for expression of an important regulator of the circadian rhythm [63]. The variability of the repeats amongst various strains of *N. crassa* leads to differences in circadian periods; TR frequencies exhibit a latitudinal cline: longer repeats (corresponding to shorter circadian rhythms) are commonly seen amongst strains collected from lower latitudes [64].

Tandem repeat variation has also been linked to differences in bird migratory patterns [65]. The avian *CLOCK* gene, a regulator of circadian patterns, contains a variable polyQ repeat in its *C*-terminus. Statistical correlation studies of the *CLOCK* gene unveil that TR variation differs between two bird species, the migratory bluethroat and the non-migratory blue tit; TR is more unstable in the blue tits population [65]. These observations suggest that variable TRs in the CLOCK gene may provide a mechanism for tuning internal clock to environmental changes in the nonmigratory blue tits. 

Such studies additionally support the beneficial role of unstable repeats, especially in diversifying gene pools, which may allow for better adaptation of organisms.

#### 5.4.2. Polyglutamine Repeats and Morphological Variation

Higher-order eukaryotes unveil another possible role for repeats. In these organisms, TRs are enriched in genes involved in determining body morphology, suggesting that unstable repeats underlie phenotypic diversity [9].

A fascinating study attributed morphological variation, occurring over short evolutionary timescales, to variations in TRs. The great diversity of morphology among dog breeds cannot merely be adequately explained by point mutations; the paper by Fondon and Garner provides compelling evidence associating variable TRs with changes in dog skull morphology occurring over a span of approximately fifty years [66]. In comparing genomic data alongside morphological data, variable TRs exist amongst different dog breeds, with two genes in particular showing quite striking results—*Alx-4* and *Runx-2*. Though the *Alx-4* gene does not contain a pure polyQ repeat but rather a PQ tract, a 51-nt deletion of this region is found in dogs with an additional rear claw (*i.e.*, bilateral rear polydactyly, a signature feature of the Great Pyrenees breed). The *Runx-2* gene, a transcription factor controlling osteoblast development, contains two polymorphic repeats—a polyQ repeat and a polyalanine repeat. Here, the ratio of glutamine to alanine repeats correlates with the degree of dorsoventral nose bend and midface length in dog breeds [66]. A further study of the *Runx-2* repeat region by Sears *et al*. suggests that the ratio of the two repeats correlates to onset of transcriptional activation [67]. The role of repeats in transcriptional regulation will be highlighted in the following paragraphs.

The morphological diversification of dog breeds over approximately 50 years, an extremely short time span from an evolutionary standpoint, is the poignant aspect of this study by Fondon and Garner. Even despite strong selection against genetic diversity as a result of domestication and inbreeding, TR involvement in controlling development and body morphology suggests that variable TR may play a critical role in the rapid evolution of dog skeletal morphology.

#### 5.4.3. Polyglutamine Repeats and Gene Transcription

A further exploration of the *Runx-2* repeats measured expression of its target and showed that mRNA of the *Runx-2* target increased with longer *Runx-2* repeats [67]. Another study, utilized CAG repeat fusions to the GAL4 transcription factor to determine changes in gene expression [68]. Gerber *et al*. provide evidence that increasing repeat number *in vitro* and *in vivo* leads to increased rates of mRNA transcription [68]. Though these studies themselves do not delve into the mechanistic aspect of increased expression, the direct effect of repeat length on gene expression sheds light on possible mechanistic effects. The following section illustrates the involvement of TR domains as sites for protein-protein interaction. 

#### 5.4.4. Polyglutamine Repeats in Protein Interactions

**Figure 6:**
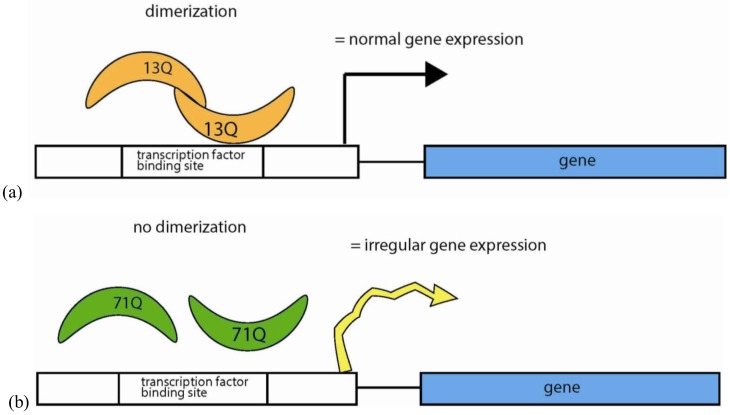
Variable glutamine (Q) TRs modulate protein dimerization. (**a**) Normal repeat length (ex*.* 13Q) of protein-interacting regions of the transcription factor TATA-binding protein allow for proper dimerization, initiating normal gene expression. (**b**) Expansion of polyQ repeats (ex. 71Q) inhibits dimerization causing irregular gene expression [69].

The expansion of polyQ tracts in the transcription factor *human TATA-binding protein* (*TBP*) gene has been shown to induce the neurodegenerative disease spinocerebellar ataxia (SCA17) [61]. Previous studies have shed light on the role of repeat expansions in conferring onset of disease, though the exact mechanism by which the polyQ repeat of TBP affects transcription has largely confounded researchers. However, Friedman and colleagues demonstrate the mechanistic role of the polyQ repeats in the transcriptional regulation of TBP [69]. TBP dimerization has previously been shown to prevent unregulated gene expression and modulate its own degradation [70]. Friedman *et al*. demonstrate that polyQ expansion inversely relates to TBP dimerization—the longer the repeat, the lower the frequency of TBP dimers (Figure 6). This indicates that the polyQ repeat tract may modulate dimerization of the protein. Additionally, TR mutations affect TBP interaction with other transcriptional factors. For example, TR expansion leads to downregulation of HSPB1 (HSP27), which encodes a small heat shock protein critical for neuronal differentiation and survival in humans. TR expansion also leads to enhanced interactions with transcription factors (e.g., the general transcription factor IIB (TFIIB)), not seen in the wild-type. This study is a clear example of a mechanistic role for variable polyQ repeats on gene function and its associated phenotypes. More studies of this type are needed to fully elucidate the role(s) of variable intragenic repeats on normal gene function and regulation. 

## 6. Conclusions

Large scale genomic studies mainly focus on Single Nucleotide Polymorphisms (SNPs) and Copy Number Variations (CNVs) as the major sources of phenotypic variation. Here, we describe studies demonstrating how tandem repeat variation can influence phenotypes and, can be considered as a third source of genetic variation.

From the evidence gleaned so far, (with the exception of repeat expansions in human diseases) TR variations do not result in deleterious changes rather in small incremental phenotypic changes (e.g., shorter or longer circadian period, lower or higher gene expression). This quantitative phenotypic variation is commonly believed to depend on complex interactions between multiple genes (*i.e.*, quantitative trait loci). However, the examples described here, show that many TRs can confer quantitative changes in phenotypes through changes in one genetic locus (coding or regulatory region). In some other cases (e.g., phase variation in pathogenic bacteria), variable TRs do function as binary switches (ON/OFF switching of cell-surface proteins in bacteria).

The formation of TRs in genomes may be a random process (resulting from polymerase slippage) [71], but once formed, some repeats might in fact be selected for. Several lines of evidence hint at such a possibility: Dinucleotide repeats, known to affect gene expression, are preferentially present proximally to transcription start sites in the human genome [72]. Some dinucleotide TRs in introns and UTRs are highly conserved (in unit sequence but not in number) in mammalian genomes [73]. These repeats may, therefore, play a conserved role, particularly since they are predominantly found in genes involved in embryonic and nervous system development [74]. The occurrence of trinucleotide repeats in six plant species was found at a 2-fold higher frequency in coding regions suggesting a selection for certain stretches of amino acids [75]. A high proportion of dinucleotide repeats has also been uncovered in the genome of multiple strains of the HIV-1 virus [76]. These examples suggest that some variable TRs are useful for genomes as they might provide a certain degree of evolutionary flexibility while other parts of the genome remain stable and unchanged [77]. The central question that returns is: Does selection favor repeats because they are variable, or is the variability merely a consequence of selection favoring some other aspect of repeat structure or function? A clear answer to that question is not possible from what we know so far, but some evidence seems to favor the possibility that variability is what makes repeats selectively advantageous. Most amino acid repeats are encoded by runs of the same DNA repeat (pure codons) which are more unstable than repeats of synonymous codons (*i.e.*, different DNA sequence but coding for the same amino acid) [5].

The appearance of TRs in genes could lead to the acquisition of a novel function and their retention could signal an evolutionary relevant path. This seems to be the case for the huntingtin protein, where the acquisition of a regulatory activity controlling neural adhesion in complex mammalian nervous systems, coincides with the appearance of glutamine repeats. Evolutionary distant heterologues of huntingtin, lacking the repeat region, fail to substitute the activity of the repeat-containing mammalian protein [78].

Taken together, tandem repeats are an underestimated, understudied source of genetic variation which, by means of their instabilities, may facilitate a rapid evolution of genes and their associated phenotypes.
